# Sapovirus, Norovirus and Rotavirus Detections in Stool Samples of Hospitalized Finnish Children With and Without Acute Gastroenteritis

**DOI:** 10.1097/INF.0000000000003493

**Published:** 2022-02-18

**Authors:** Oskari Pitkänen, Jukka Markkula, Maria Hemming-Harlo

**Affiliations:** From the * Vaccine Research Center; † Tampere Center for Child Health Research, Arvo Ylpön katu 34, University of Tampere, Finland; ‡ The Pediatric Research Center, Biomedicum Haartmaninkatu 8, University of Helsinki, Finland

**Keywords:** epidemiology, pediatric, communicable diseases

## Abstract

**Background:**

Sapovirus, norovirus and rotavirus are major causes of childhood acute gastroenteritis (AGE) globally. Asymptomatic infections of these viruses have not been extensively studied.

**Aim:**

To examine the prevalence and the genetic variations of sapovirus, norovirus and rotavirus in children with and without symptoms of AGE.

**Methods:**

We collected 999 stool samples from children under 16 years old from September 2009 to August 2011 at Tampere University Hospital, Finland. In total 442 children (44%) had symptoms of AGE and 557 patients (56%) had acute respiratory tract infection (ARTI) only. Samples were examined for sapovirus, norovirus and rotavirus using reverse transcription-polymerase chain reaction and the positive amplicons were sequenced.

**Results:**

Totally 54% and 14% of the patients in AGE and ARTI groups, respectively, tested positive. All viruses were more frequently detected in AGE patients than in ARTI patients (norovirus, 25% vs. 7.2%, respectively; rotavirus, 24% vs. 6.1%; sapovirus, 5.2% vs. 1.4%). In ARTI patients, the cases were seen most frequently during the first two years of life. Norovirus was the most detected pathogen in both groups with genogroup GII covering ≥97% of norovirus strains. Sapovirus was mostly detected in children under 18 months old without predominating genotype. Rotavirus was often detected after recent rotavirus vaccination and 18% and 88% of the strains were rotavirus vaccine-derived in AGE and ARTI groups, respectively.

**Conclusions:**

We showed that the most common viruses causing gastroenteritis in children may be found in the stools of an asymptomatic carrier which may function as a potential reservoir for AGE.

Caliciviruses, especially norovirus and sapovirus, together with rotaviruses are major causes of childhood acute gastroenteritis (AGE). While caliciviruses are important causes of nosocomial and institutional outbreaks, rotavirus is still showing the highest burden of severe diarrhea in developing countries.^1^ They all spread through the fecal-oral route and mainly cause vomiting and nonbloody diarrhea.^2–4^ The incubation period of rotaviruses has been described slightly longer compared to caliciviruses.^5^

Norovirus may be detected in stools from several weeks to over 2 months after infection. Hence, asymptomatic transmission of norovirus is common,^6^ and similar to that in symptomatic infections showing notable individual variation.^7^ It has been estimated that approximately every third norovirus infection is subclinical in adults.^8,9^ Further, the number of asymptomatic norovirus detections may be 2-fold in children compared to adults.^10^

The prevalence of sapovirus ranges from 4% to 19% and 3% to 15% in outpatients and hospitalized children with AGE, respectively.^11–14^ Viral shedding of sapovirus has been described to last from 1 to over 5 weeks after infection^3,15^ and asymptomatic infections have been detected in even up to 20% of children.^1^ A recent Danish surveillance study showed that every fifth child attending day care shed sapovirus at some point over the period of 12 months and more than half were asymptomatic at the time of testing.^16^

The proportion of asymptomatic rotavirus infections has previously been reported lower (1%–2%) in comparison to caliciviruses,^6,17^ and shedding of wild-type rotavirus has been reported up to weeks after infection.^18^ In Finland, a live human-bovine reassortant rotavirus vaccine, RotaTeq (Merck & Co., Kenilworth, NJ), was taken into the national immunization program in September 2009 and has been administered since on a 3-dose schedule at the ages of 2, 3 and 5 months. Shedding of vaccine-derived rotaviruses were reported already in the early clinical studies and more recent studies have confirmed shedding of RotaTeq strains even as common as 90% a week after the first dose.^19–21^

The aim of this prospective study was to examine the prevalence of symptomatic and asymptomatic circulation of noroviruses, sapoviruses and rotaviruses in children in a hospital-based setting.

## MATERIALS AND METHODS

### Samples

Stool samples were collected at Tampere University Hospital, Finland, from September 2009 to August 2011, after the introduction of national rotavirus vaccination in Finland in September 2009. The hospital is the pediatric referral center for the Pirkanmaa Hospital District covering approximately 550,000 residents. The study followed the regulations in the Declaration of Helsinki and was approved by the Ethics Committee of Pirkanmaa Hospital District, Finland. The criteria for AGE were 3 or more loose stools or 2 or more vomits or 1 loose stool and 1 vomit in 24 hours before or during the hospital admission, in connection with another clinical diagnosis. All the children under 16 years of age with AGE who visited the emergency department or were admitted as inpatients were eligible to take part in the study. The other group was formed by children under 16 years of age admitted to the ward with acute respiratory tract infection (ARTI) and showed no AGE symptoms. Written informed consent was signed by a parent or a legal guardian by the time of original study admission. Clinical study methods have been described in more detail previously by Paloniemi et al^22^

Statistical analysis was performed using the *χ*^2^ and Fisher exact test or Independent-samples *t* test in IBM SPSS Statistics 27 program (IBM Corp., Armonk, NY). *P* values below 0.05 were considered as statistically significant.

### Laboratory Methods

After collection, stool samples were stored in freezers at −°20°C. Viral RNA was extracted using Qiagen QIAamp Viral RNA Mini Kit (Hilden, Germany) according to the manufacturer’s protocol. The samples were examined for rotaviruses, noroviruses and sapoviruses using reverse transcription-polymerase chain reaction and nucleotide sequencing as described previously.^23,24^ The re-extracted samples were sequenced using the BigDye Terminator v3.1 Cycle Sequencing Ready Reaction Kit (Applied Biosystems, Foster City, CA) on an ABI 3500XL Genetic Analyzer (Thermo Fisher Scientific, MA). Results were analyzed with Sequencher 4.10.1 (Gene Codes Corp Inc., Ann Arbor, MI). The obtained sequences were compared to previously published sequences from the nucleotide database in GenBank (National Center for Biotechnology Information, Bethesda, MD), using the basic local alignment search tool, and from the foodborne viruses in Europe Network, using the norovirus automated-genotyping tool.^25^

## RESULTS

A total of 1610 patients were eligible for the study. Of those, 999 children provided a stool sample, of which 58% and 42% were obtained during the study seasons of 2009–2010 and 2010–2011, respectively. Of the final study population of 999 children, 442 (44%) had symptoms of AGE (32% from the emergency department and 68% from the ward) and 557 (56%) were admitted with symptoms of ARTI only. The median age of all children was 1 year 5 months (range 0 months–15 years 7 months) and 62% of the children were male. Of all obtained samples, 304 (30%) were positive for 1 or more of the studied gastroenteritis pathogens: 151 were positive for norovirus, 140 for rotavirus and 31 for sapovirus. Of the 304 positive samples, 87 (29%) were collected from patients with AGE at the ward, 140 (46%) were collected from AGE patients in the emergency room and the rest, 77 samples (25%), were collected from children with ARTI. There were 12 mixed infections with rotavirus and norovirus and 5 mixed infections with rotavirus and sapovirus. Mixed infections with norovirus and sapovirus were not detected.

### AGE Patients

In the 442 children with AGE, one or more of the studied pathogens was detected in 54% of the samples, 111 (25%), 106 (24%) and 23 (5.2%) were positive for norovirus, rotavirus and sapovirus, respectively. The patients had a median age of 1 year and 3 months (range 0 months–15 years and 7 months). There were 12 (2.7%) mixed infections: 7 cases with norovirus and rotavirus (3 wild types and 4 rotavirus vaccine-derived) and 5 cases with rotavirus (3 wild types and 2 rotavirus vaccine-derived) and sapovirus without association to specific sapovirus genotypes.

### ARTI Patients

In the 557 children with ARTI, studied pathogens were found in 15% of the samples; norovirus was detected in 40 (7.2%), rotavirus in 34 (6.1%) and sapovirus in 8 (1.4%) cases. The median age of ARTI children with positive AGE virus detection was 7 months (range 0 months–7 years). There were 5 cases with mixed infection of norovirus and rotavirus (1 wild type and 4 rotavirus vaccine-derived). In ARTI children from 0 to 5 months, 6 to 11 months and 12 to 17 months of age, 24%, 15% and 13% were positive for tested viruses, respectively, whereas in children from 2 to 5 years of age 9.2% were positive. The asymptomatic calicivirus-positive cases did not show a statistically significant correlation with the age of the patient (*P* = 0.48, Independent-samples *t*-test).

### Norovirus

**FIGURE 1.**
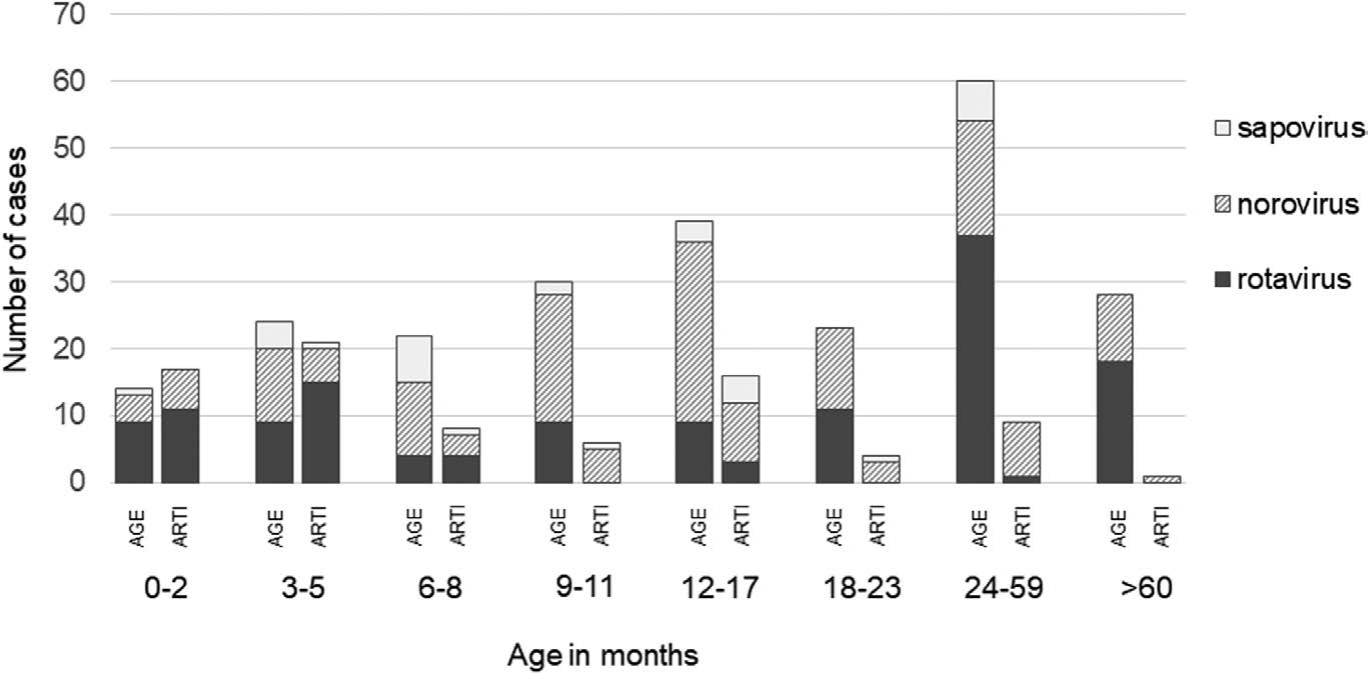
Distribution of the detected gastroenteritis virus positive cases by age and symptoms.

Norovirus was the most detected virus in both symptomatic and asymptomatic children. The proportion of the asymptomatic cases of all norovirus infections was the highest, 42%, in children under 6 months of age, and remained at 28% in children under 18 months old, before declining (Fig. 1). In all norovirus positive samples, genogroup GII was the most prevalent finding with 108 of 111 cases in AGE and 39 of 40 cases in ARTI patients. Further, 62% and 51% of these presented genotype GII.4 New Orleans 2009 in AGE and ARTI groups, respectively. Genotypes GII.P21 and GII.P33 comprised 13% and 5% of cases in the AGE group, respectively, but were not detected in the ARTI group. Genotyping of the capsid region C was successful in 133 norovirus-positive samples. The genotyping results are depicted more in detail in Table 1.

**TABLE 1.** Genotyping Results of the Detected Rotavirus, Norovirus and Sapovirus Strains in All Patients

Virus	Group		Summary (n)
	AGE (n = 442)	ARTI (n = 557)	999
Rotavirus (% of samples)	106 (24%)	34 (6.1%)	140 (14%)
By G-genotypes	39 × G1 30 × G4 15 × RotaTeqG1 9 × G2 6 × G9 2 × RotaTeq G1 + RotaTeq G4 1 × G3 1 × G1+G3 1 × G3+G9 1 × RotaTeq G6 1 × RotaTeq G1 + RotaTeq G3	27 × RotaTeq®G1 2 × RotaTeq®G6 1 × G1 1 × RotaTeq G1 + RotaTeq G6 1 × G4 1 × G9	
Vaccine-derived (% of rotavirus positive)	19 (18%)	30 (88%)	49 (35%)
Norovirus (% of samples)	111 (25%)	40 (7.2%)	151 (15%)
Genogroup GI (% of the norovirus positive)	3 (2.7%)	1 (2.5%)	4 (3%)
GI.4	2	–	2
GI.PNA1 (GI.Pb)/GI.6	–	1	1
GI.3	1	–	1
Genogroup GII (% of the norovirus positive)	108 (97%)	39 (98%)	147 (97%)
GII.P4 NO2009*	63	12	75
GII.P21 (Pb)/GII.3	14	–	14
GII.P7/GII.6	12	3	15
GII.P4 NO2009/GII.4 CNA†	2	4	6
GII.P4 A2007‡/GII.4 NO2009	3	–	3
GII.P33 (Pg)/GII.1	3	–	3
GII.P4 CNA/GII.4 NO2009	2	–	2
GII.P31 (GII.Pe)/GII.4 NO2009	–	2	2
GII.P4 A2007/GII.4 CNA	–	2	2
GII.P7/GII.14	1	1	2
GII.P7	1	–	1
GII.P31 (GII.Pe)/GII.4 S2012§	–	1	1
GII.P31 (Pe)/GII.4 O2007¶	1	–	1
GII.P33 (Pg)/GII.12	1	–	1
GII.P33 (Pg)/GII.14	1	–	1
CNA†	4	14	18
Sapovirus (% of samples)	23 (5.2%)	8 (1.4%)	31 (3.1%)
Genogroup GI (% of the sapovirus positive)	10 (43%)	7 (88%)	17 (55%)
GI.1	8	2	10
GI.2	1	1	2
GI.4	1	4	5
Genogroup GII (% of the sapovirus positive)	12 (52%)	1 (13%)	13 (42%)
GII.1	10	–	10
GII.3	1	1	2
GII.4	1	–	1
Genogroup GV (% of the sapovirus positive)	1 (4.3%)	–	1 (3.2%)
GV.2	1	–	1

AGE, acute gastroenteritis; ARTI, acute respiratory tract infection.

* NO2009 = New Orleans 2009.

† CNA = Could Not Assign.

‡ A2007 = Apeldoorn 2007.

§ S2012 = Sydney 2012.

¶ O2007 = Osaka 2007.

### Sapovirus

Sapoviruses were detected in all age groups, but 24 (77%) cases were detected in children under 18 months of age. Genogroup GII was more associated with AGE (Table 1), but no statistical significance was found between the presentation of gastroenteritis symptoms and the sapovirus genogroups (*P* = 0.12, *χ*^2^). Asymptomatic sapovirus infections were not detected in children older than 2 years of age.

### Rotavirus

Rotavirus was the leading cause of AGE in children between 0 and 6 months and over 2 years of age. The prevalence of rotavirus in ARTI patients was highest in children under 6 months of age (26 positive in 142 children, 18%), where the asymptomatic infections accounted for 59% of all rotavirus infections. In children over 2 years old, only 1 asymptomatic rotavirus infection was detected (Fig. 1). There were 19 (18%) and 30 (88%) rotavirus vaccine-derived rotavirus positive cases in AGE and ARTI groups, respectively. RotaTeq G1 was the most common strain in children with ARTI and frequently detected also in the AGE group.

## DISCUSSION

We conducted this prospective hospital-based study on the epidemiology of 3 major gastroenteritis viruses; norovirus, sapovirus and rotavirus, in children diagnosed with AGE or ARTI soon after the beginning of universal rotavirus vaccination. The results showed that all studied viruses may be found in stools of an asymptomatic carrier which may function as a potential reservoir for AGE and therefore may increase viral transmission and the number of outbreaks.

The detection rate of AGE viruses in the ARTI group was highest in newborns, steadily declining towards older children. As expected, rotavirus was often detected in the stools after rotavirus vaccinations. Also, noroviruses and sapoviruses were most often detected in younger age groups. The virus detection rates are supported by studies from other developed countries, such as Denmark, Spain and the USA.^26–28^

Norovirus was the most prevalent of the studied viruses in the whole cohort but also in both subgroups. The norovirus detection rate of 7.2% in the asymptomatic is similar to previous publications.^28–30^ Norovirus GII.4 caused the majority of norovirus infections also globally during the study period.^31^ Close to our study period, norovirus genogroup GII was more prevalent (92%) than GI (8%) in the USA, with GII.4 New Orleans as the most common genotype both in AGE and in healthy controls..^28^ In Latin America, norovirus GII was detected in 67%–100% of the asymptomatic infections but over 90% of these were non-GII.4 genotypes.^32,33^

Sapovirus was found in all age groups, but notably, most of the positive samples were detected in children under 18 months of age and no asymptomatic cases were detected in children over 2 years old. In comparison to our result of 1.4%, recent publications from Bangladesh, Peru and USA detected sapovirus in 2.6%–9.7% of stool samples from asymptomatic children.^15,28,34^ We did not detect a significant difference between the presentation of AGE symptoms and the sapovirus genogroups. In the USA in 2012, sapovirus GI genogroup was the most detected in the control group, and genotypes GI.1 and GII.1 were in the 3 most detected genotypes in AGE patients.^28^ On the contrary, in Peru, GI strains were found more frequently in symptomatic infections with statistical significance, and GII viruses were more commonly detected in asymptomatic infections.^15^

RotaTeq vaccine was implemented as part of the National Immunization Program at the same time with the beginning of this study and the coverage of the rotavirus vaccination was very high at 92%–93% already in 2011.^35^ The number of rotavirus infections, especially without gastroenteritis symptoms (ARTI group), peaks at 0–6 months of age and the genotyping results showed that this is highly likely due to rotavirus vaccine shedding, whereas the wild-type rotaviruses were detected among the older children. Rotavirus vaccinations explain the notable difference in age distribution between the symptomatic (15 months) and the asymptomatic children (7 months).

All the studied viruses show considerable variation in peak levels and the duration of virus shedding.^7,36,37^ A recent Dutch study suggested that prolonged shedding after AGE accounted for about 25% of all virus detections.^6^ However, in Japan, only 4.5% of asymptomatic sapovirus infections were due to viral shedding after the latest AGE.^38^ Shedding cannot be ruled out in this study as the guardians were asked about the symptoms present only in the last 24 hours before hospitalization. The viruses are likely to have been transmitted to the infants from their family members, but eventually, older age and siblings have been recognized as protective factors against the shedding of norovirus and sapovirus likely because of the earlier exposure to the pathogens^16^ To support this, in our study, the number of asymptomatic AGE virus detections did not increase, but rather decreased, by age cohorts (Fig. 1). However, the immune response to norovirus seems very type-specific and might not avert the following infections with a heterologous virus.^39^ This has also been detected regarding sapoviruses.^38^ The varying definitions of AGE between published studies made the results harder to compare. Further, quantitative PCR methods are needed to examine the role of viral load in relation to the appearance of clinical symptoms.

## CONCLUSIONS

Sapovirus, norovirus and rotavirus were regularly detected in asymptomatic children especially under 2 years of age and the number of asymptomatic AGE virus detections decreased by age. Norovirus was the most common of the studied pathogens in symptomatic and asymptomatic infections with a slightly different genotype pool between the groups. Wild-type rotaviruses were mostly seen in older, symptomatic, unvaccinated children whereas the administration of rotavirus vaccines was seen to cause a peak in the asymptomatic rotavirus detections in children under 6 months of age. Sapovirus was sporadically seen and there was a notable difference in presenting genotypes between the groups.

## ACKNOWLEDGMENTS

We thank Professor Timo Vesikari MD and the personnel of Vaccine Research Center (Stina Gröhn, Sanna Kavén, Nina Koivisto, Marjo Salminen, Marjo Salonen).
